# Self‐Giving and Reflections on Life Extension: How Love Might Shape the Choice of Whether to Live Past a Natural Human Lifespan

**DOI:** 10.1111/bioe.70024

**Published:** 2025-08-13

**Authors:** Andrew Moeller, Ann‐Marie Shorrocks, Keith Lemna

**Affiliations:** ^1^ University of Oxford Oxford UK; ^2^ Saint Meinrad Seminary and School of Theology Saint Meinrad, Indiana USA

**Keywords:** human lifespan, life extension, love, meaning, purpose

## Abstract

Drawing upon a deprivationist account of the badness of death, Ingemar Patrick Linden advocates for a hypothetical state called “contingent immortality.” The future Linden champions is one in which every person would be able to live for as long as they would like, save for events like accidents or murder. We recognize Linden's foundational claims in defense of contingent immortality as weighty and reasonable, but consider whether there are defensible reasons to forgo the inhibition of aging and living well past a natural human lifespan. Drawing partly upon the work of Carter Snead and Alasdair MacIntyre, we outline the nature of self‐giving love, how love provides for many persons a measure of meaning and purpose in life, and the ways in which given and self‐imposed limitations can help imbue our actions as embodied beings with a particular and richer sense of that meaning and purpose. We conclude that love provides defensible grounds as to why one might reasonably choose to accept our shared human identity as creatures with naturally bounded lifespans. This conclusion takes into account both the benefits and costs of love, especially in light of our existence as embodied beings.

## 1 Introduction

In his thoughtful book, *The Case Against Death*, Ingemar Patrick Linden advocates for a hypothetical state called “contingent immortality.” That is, he writes in part to foster public opinion toward pursuing a future wherein medical science could empower people to live as long as they would like.^1^ Following a deprivationist line of thought,^2^ his general case against death is that it

robs a person of all the goods one would have enjoyed if one had continued to live. Among these goods are the mental skills, valued experiences, and personal relationships built up over the course of a life.

Linden also laments the many ailments associated with the aging process; and is understandably ardent in his analysis. He describes death as a “horrific evil,” adding that “nothing is more important than preventing good people from dying” [3].

Linden is noncommittal as to how contingent immortality might be achieved, but he does imagine a pill for the inhibition of aging. We will consider this option, and presume that one would choose to inhibit the aging process when they reach their mid‐20s. That is when the brain completes its development [4], and is around the age when a person reaches the peak of their natural physical fitness.^3^ Linden does not settle on a specific number as desirable, but within his moral framework, the correct moral decision for the vast majority of people would be to inhibit aging and live well past the natural human lifespan (presumably for many hundreds or even thousands of years). In an imagined future free from the ailments associated with aging, Linden concludes that he would choose to die when he deemed it superior to the available goods made possible through continued existence. Importantly, he also disputes that living well past an average human lifespan would necessarily produce an existential malaise or result in undesirable social outcomes like overpopulation and related resource scarcity (we will discuss these claims later in this article). Thus, for Linden, a naturally bounded lifespan is part of our shared human identity, but it is a vain and loathsome trait [3, pp. 17–20, 167–168, 105–109, 20, 203, 40, 28, 12–18].

In this article, we will consider whether there are defensible reasons for limiting oneself to a natural human lifespan. Drawing upon various works of literature, as well as the philosophical thought of Carter Snead and Alasdair MacIntyre, we will outline the nature of self‐giving love (referred to hereafter as “love,” unless otherwise stated), how love provides for many persons a measure of meaning and purpose in life, and the ways in which given and self‐imposed limitations can help imbue our actions as embodied beings with a particular and richer sense of that meaning and purpose. We conclude that love provides defensible grounds for why a person might reasonably choose to accept our shared human identity as creatures with naturally bounded lifespans.^4^ This conclusion takes into account both the benefits and costs of love, especially in light of our existence as embodied beings.^5^

### 1.1 On the Possibilities of Life Extension and Living Well as Embodied Beings

The oldest verified person on record, Jeanne Calment, lived to 122 years. Disagreement persists as to whether that age represents a rough, upper‐limit to the natural human lifespan (meaning a lifespan limited by the natural aging process).^6^ Even if such a limit is disputed, there is broad agreement that the future Linden imagines will never come about through lifestyle changes like caloric restriction or increase in exercise. That will necessitate breakthroughs with biotechnologies—like cellular reprogramming for the reversal or inhibition of aging.^7^ The aim of cellular reprogramming is taking fully developed adult cells and essentially turning back the clock on their development.

Although a thorough discussion of these biotechnologies is beyond the scope of this article, we remain unconvinced as to the feasibility of all the prominent proposals, including cellular reprogramming, for enabling human beings to live as long as they would like.^8^ This is partly because we are integrated and unified creatures. The complex nature of our given embodiment makes it correspondingly and exceedingly complex to inhibit aging without significant and detrimental side‐effects. For example, as an expert in cancer cell biology, one author of this paper (A,M, Shorrocks) assesses that striking the balance between partially reprogramming all adult cells to a “more youthful state,” without completely erasing their identity and risking uncontrolled cell growth and therefore cancer development poses a difficult and likely insurmountable challenge, especially given the many different kinds of cells in the human body.^9^ For the purposes of this article, we will nevertheless grant a future in which all people can make a choice to live for as long as they might like (save for events like accidents and murder).

There are several broad options within Linden's imagined future that are relevant to our consideration of life extension. The first is a natural human life cycle/lifespan. The second is Linden's hypothesized contingent immortality, in which we have suggested a person would inhibit aging around the age of 25. It is implied they would thereafter be impervious to age‐related ailments, and presumably also death from any disease.^10^ This would not be the same as possessing an indestructible body (one could still die in a car crash, for example), nor the same as an immortality defined in a negative sense as the inability to die. Given the contingent immortality framework, a person could conceivably decide at an indeterminate future date, when death seemed preferable to them, to commit suicide or embrace the natural aging process. A third and related option is that a person could choose to inhibit aging, but still proactively plan to limit their years on this earth. For example, one might decide for themselves, before inhibiting aging, to live to 200 or 300. We will consider that third option at the end of this article, but will focus primarily on contrasting the first and second options.

Due to the complexities involved in inhibiting aging, we will anticipate the achievement of contingent immortality is hundreds of years from us. With that consideration in mind, we will specifically compare the choice of contingent immortality against the option of a natural human lifespan in which someone dies at 100 years old. We have chosen 100 because Linden draws upon a study by the United Nations that predicts the average human lifespan in the “developed world” will reach approximately that number by 2300 [3, p. 1]. As an inherent part of our second option, natural aging will occur—but we also assume that medical advancements and palliative care will similarly have advanced such that a person will be significantly less likely to experience intense and debilitating ailments for long periods of time.

As a final preparatory note, we hold aging to be a constituent part of our shared nature as embodied, integrated, and unified human creatures—who partake in a particular and given lifecycle. We similarly hold mortality to be a constituent trait, or part of our shared humanness. Furthermore, we also exist as embodied beings who age and die within webs of human communities–to be human is to be a being‐in‐communion with other humans.

One of course would not cease to be human if death (and the aging process that often precedes it, and which death is a culmination of) could be largely subverted. But might such a subversion represent an escape from the aspects of the givenness of our shared nature as embodied beings (who move and exist within, and experience the world as such) that are integral to living well as individuals and members of communities?^11^ This is not to automatically conflate the givenness of aging and death with the good,^12^ meaning here the superior way of living in the world. But it is to start with an openness toward the idea that the givenness of aging and death to us as embodied beings might serve some significant ends relating to the holistic pursuit of living well in the world as individuals and members of communities. In this article, we argue that such ends include providing a particular and rich sense of meaning and purpose to acts of love; relief from the rigorous work of love, a sense of orientation as it relates to how far one has to run in the “race” of love, and a bulwark against antagonism and instrumentalization within the context of loving relationships.

## 2 The Nature of Self‐Giving Love

When offering up our definition of love, we are not arguing for or against the desirability of other descriptions or definitions of love, and we are not reducing all other understandings to accord with our definition. Furthermore, we are not providing an exhaustive treatment on what it means to love. We instead use the term as shorthand for a description of a way of relating to other human beings, often in contexts like kinship, romantic relationships, and long‐term friendships, by which many people find significant meaning and purpose in life. Drawing upon a long history of thought and reflection in literature and philosophy, itself significantly influenced by accounts of the life and self‐sacrifice of Jesus Christ in the Gospels, we use the term to refer to an act, orientation, or lifestyle of self‐giving in the service of the good of another, with the addition that the person expressing love toward another intentionally understands themselves to be in a favorable position.

When speaking of the nature of self‐giving love, what might be called extraordinary acts are often held up as examples. In his 20th century novel *Life and Fate*, Vassily Grossman tells the story of a woman sent to a German concentration camp. When given the chance to save herself, she instead chooses to accompany a young, scared boy into the gas chambers—so as to make his last moments on earth more bearable. Grossman describes these kinds of extraordinary expressions of love elsewhere in the novel as ones of “senseless kindness” [8].

However, in his recent book on the body and bioethics, Carter Snead recounts some simpler, more quotidian acts of love—such as a child caring for an aging parent. Drawing on the work of Alisdair MacIntyre, Snead asserts that, “as living bodies in time, we are vulnerable, dependent, and subject to natural limits … Thus, both for our basic survival and to realize our potential, we need to care for one another.” He clarifies that this care entails making the good of others our own good, “without the demand for or expectation of recompense,” adding also that “by virtue of our existence as embodied beings in time, we are made for love and friendship” [9]. Drawing upon the work of twentieth‐century novelist Dorothy Sayers, it can be said that even if one disagrees with Snead's assessment that humans are made for the expression and reception of love, it still holds true that experientially, many people have found that leading a life oriented toward love to be a meaningful and worthwhile endeavor [10].

### 2.1 A Glad Presence

Based on the writings of Grossman and Snead, the favorable position of one being loved is perhaps understood more readily. Within the context of human relationships, the one receiving love often obtains tangible benefits (such as a child receiving food and clothing from a parent). But the good is also found through the glad presence of another. Matthew Liao defines love as directed toward a child as such:

to love a child is to seek a *highly intense interaction* [emphasis my own] with the child, where one values the child for the child's sake, seeks to bring about and maintain physical and psychological proximity with the child, seeks to promote the child's well‐being for the child's sake, and desires that the child reciprocate or, at least, respond to, one's love [11].

As Liao highlights, the experience of the glad presence of another (e.g., expressions of warmth) is central to the wellbeing of children [11, Ch.4]. Common experience and research suggest that adults likewise benefit [12]. We find a particularly striking example of this in literature, with Victor Hugo relaying in *Les Misérables* something of the latter life of Bishop Myriel—who previously rescued Jean Valjean with his own act of self‐giving. We are told the following about the bishop's final years, when he became blind and was cared for by his sister:

Let us remark by the way, that to be blind and to be loved, is, in fact, one of the most strangely exquisite forms of happiness upon this earth, where nothing is complete. To have continually at one's side … a sister … who is there because you need her and because she cannot do without you; to know that we are indispensable to a person who is necessary to us; to be able to incessantly measure one's affection by the amount of her presence which she bestows on us … One sees nothing, but one feels that one is adored. It is a paradise of shadows [13].

Hugo pictures the bishop's happiness (defined here as a deep‐seated delight) as flowing from the reception of necessary care, but also in enjoying the relational presence of his sister. Part of that enjoyment, according to Hugo, was found in the bishop knowing that she deeply valued him; that she delighted in him.

To love, however, is also to find oneself in an advantageous position [14]. Here we would not conflate the benefits entirely with self‐reported accounts of happiness—often defined in contemporary terms as a general sense of psychological contentment. There are many ways to orient oneself toward service of the good of others, with one possibility being the raising of children. Adults without children generally report higher levels of happiness than parents with children still in the home.^13^ One reason for this might be the constant and significant self‐giving required within the framework of a loving orientation toward a child. Despite lower levels of self‐reported psychological contentment, many parents still indicate that raising children provides them with significant meaning and purpose.^14^

It is in reference to meaning and purpose that we chiefly include as part of our definition of love that the one expressing love consciously or willingly regards themselves as in an advantageous position. One might then be eager to serve another because they understand it as deeply significant that the person whom they value flourishes, and they find meaning and purpose in witnessing and playing a part in that flourishing (many loving relationships are of course reciprocal in nature).^15^ There is also the simple relational presence of another that a person expressing love may experience as a source of deep meaning—such as a parent with a newborn.

## 3 Love and Limitations

MacIntyre suggests a human tendency “to conceive of ourselves and imagine ourselves as other than animal,” or as he famously puts it: to be “forgetful of our bodies” [17].^16^ And following the thought of MacIntye and Snead, we might remind ourselves that our limited, bounded form is the means by which we can express and bestow love. With our definition of love in mind, we can in fact think of no human way of expressing love that is not interwoven with at least one of our shared, natural limitations as human beings.^17^ When we are consciously aware of them, the limitations at play can also help imbue acts of service in the good of another with a particular and richer sense of meaning and purpose.^18^

Consider the context of a professor with her students. The professor may express an appropriate orientation of love in that, conscious of her limited number of years on earth, she at some point in time chose the profession of teaching (out of many other possibilities) partly to serve the good of students. She could have then counted it as a benefit to spend thousands of her limited lifetime of hours to earn her credentials. And she may continually choose, for example, to spend the limited time and energy she knows she has in any given day in offering excellent teaching, providing office hours, taking on committee memberships, etc., at least in part for the good of her students. Awareness of such limitations in terms of years on this earth and energy to expend in any given day could then supply a specific and richer sense of meaning and purpose to the choice of becoming a professor, as well as the daily practices associated with that vocation.^19^ Additional limitations relevant to one's profession and the expression of love might include the decline in strength and stamina that accompanies aging, the fact that we can only be physically present in one location at any time, and also physical vulnerability (that we can be injured and experience pain may be especially relevant to vocations like firefighting or policing).

## 4 The Costs and Benefits of Loving as Embodied Beings

Thus far, much of what we have stated could be read in support of Linden's case against death. That is, if it is agreed that loving and being loved can be a source of meaning and purpose, death could be construed as an enemy because it severs loving relationships and prevents further self‐giving and receiving.^20^ Furthermore, even if death were entirely removed from consideration, consciousness of other natural limitations could still enrichen the experience of self‐giving.^21^ We will return to these ideas below. But for now, Linden's claim might still hold true, in that

true courage in the face of mortality is not to pretend that it is a blessing, or in other ways seek to tame death; rather, true courage is to accept that if death is the end then this is a gruesome fact and that therefore the human condition, while wondrous and enjoyable, is fundamentally tragic [3, p. 18].

Linden further suggests that the maximum human lifespan is evolutionarily contingent [3, pp. 1–2]. Supposedly, humans could have found themselves with a natural maximum lifespan of 50 or 500. Hence, if contingent immortality were made readily available, a choice by someone with a generally good life (and who can expect in the future a generally good life) to accept the universal aging process and a 100‐year life on the grounds that the human lifecycle is “natural” would be arbitrary and a false conflation of the given with the good.^22^

As mentioned, Linden also rejects arguments against life extension that center around existential or moral benefits (such as death teaching us to prioritize our lives, or the interpretation of Leon Kass as to why death can be considered the “mother” of beauty), as well as those that highlight concerns over resource allocation, overpopulation, social security solubility, etc. He believes overpopulation will likely not be a problem for several reasons, including that: (1) in regions where life expectancy increases, people generally choose to have fewer children; (2) people will continue to die in Linden's imagined future; (3) if needed, there might be ways to randomly limit the number of couples who wish to have large families from also having access to life extension technologies [3, pp. 151–157].

The natural conclusion is that there are no defensible reasons as to why a person with a generally good life would not choose to live many hundreds of years past a natural human lifespan [3, pp. 16–18, 201–207]. We do not accept many of Linden's claims against the arguments listed above, but engagement with most of them falls outside the scope of this article. Importantly, all we set out to do below is offer at least one reason as to why a person with a generally good life, and who ascribes to what Linden calls a “normative” understanding of human nature (i.e., that it is bad to have destructive desires; which would generally include sickness and suffering, as well as the forfeiture or frustration of goods caused by death), might accept as sensible [3, p. 17], even if they themselves would act differently in the same circumstances.

### 4.1 Self‐Imposed Limitations

Here, we return to our discussion of the value of limitations, and particularly our self‐awareness of those limitations, in relation to love. Thus far, we have spoken of given or natural limitations. In that sense, awareness of the given limitation of a naturally bounded human lifespan can help imbue expressions of love with a particular and rich experience of meaning and purpose. That is a central reason as to why one expressing love is right to consider themselves to be in an advantageous position. But there are also chosen or self‐imposed limitations that can similarly provide a rich and particular sense of meaning and purpose. For example, billions of people across varying religions hold monogamous marriage to be a deeply meaningful institution that is in some sense divinely ordained. Others assess it as a social construct [20].

Of course, a habit or custom that is a social construct can still be deeply meaningful to many people. For example, the giving of flowers or a card on Valentine's Day. With that in mind, let us assume the arbitrary/social construct position as it pertains to marriage. Reasons for marrying could still include companionship and securing long‐term help with the raising of children. But then, why limit yourself to marrying one person? Would 5 partners not provide more companionship and prove to be a greater help in raising kids? In response, it could be said that choosing to live a circumscribed or self‐limited life, in this instance making the choice to marry only one person (even if it may be an arbitrary decision), is a means by which many people find a unique and rich kind of meaning and purpose in life. Especially so within a framework of a loving orientation toward that other person.

By adopting such an orientation, one might be saying: “I will love you, and only you, in a particular and special way. By sharing a home together. By sharing children. By supporting each other's hopes and dreams each and every day. I will focus on serving your good in a particular and special way, in comparison to all other people.” Another, related reason for choosing to marry one person returns us to natural limitations—which cannot always be conflated with waning capacities linked to the natural aging process. In this instance, it might be said: “I have a desire to love. But I recognize that I am a limited person and only have so much capacity for self‐giving. The thought of expressing such an intense and enduring kind of love towards 5 people seems to me more a burden than advantage.” Thus, a self‐limiting choice, such as to marry one person, could take into account both the costs and benefits of love. In a similar way, a choice to limit oneself to a natural, 100‐year human lifespan could be considered arbitrary (in that one could believe that lifespan to be evolutionary contingent). Yet, the choice may still be a source of significant meaning and purpose, and such a choice might also reasonably take into account both the benefits and costs of love to us as embodied beings.

### 4.2 Lucretius and Life as a Banquet

Martha Nussbaum, in dialogue with John Martin Fischer, considers an analogy by Lucretius that might be employed against life extension. As summarized by Nussbaum, Lucretius taught that

life is like a banquet: it has a structure in time that reaches a natural and appropriate termination; its value cannot be prolonged far beyond that, without spoiling the value that preceded. Mortals should therefore not strive to prolong their lives indefinitely, since this will just spoil the pleasure of the life they have.^23^

Taking into account natural human limitations, here we reimagine the entirety of life as a kind of banquet for our own purposes. Given our definition of love, we could picture ourselves as both *participants* and *servants* at the banquet. This is not to conflate a life of love with drudgery; but with meaningful and often rigorous mental, emotional, and physical work in the service of others. As previously alluded to, that kind of work can prove rigorous regardless of biological age (e.g., the raising of children, any job entailing manual labor, or consoling a despondent friend).

Importantly, the choice of contingent immortality presumably entails one selecting to inhibit the aging process around the age of 25. As such, one needs to make informed presumptions about how their life will unfold. Given that scenario, one may then be able to reasonably anticipate they will be ready for death at 100, at that point believing it likely they will experience a general sense of both satisfaction and desire for respite as a result of having spent their lives with an orientation toward the good of others.

Building on the above, and relating to the often‐referenced idea that death helps to provide a valuable kind of narrative structure to one's life,^24^ a 100‐year time span could provide an important reference for the amount of anticipated work remaining in relation to spending one's life in service to others. Reflecting on the life spent in self‐giving that is ascribed to her main character in *Middlemarch*, George Elliot says this:

Her [Dorothea's’] full nature, like that river of which Cyrus broke the strength, spent itself in channels which had no great name on the earth. But the effect of her being on those around her was incalculably diffusive [22].

Switching to a comparison of living a life of love to the running of a race, it would be a relief to Dorothea and most runners to know how many miles are set before them in any particular contest. In contrast, it would be disorienting and disheartening to run a race with no obvious end. So then, even if other human limitations can richly imbue self‐giving with meaning and purpose, it does not necessarily follow that living an indefinite period past 100 years old would be desirable.

An aging body can provide another kind of important reference point, in a sense demonstrating how a person has poured themselves out in love. To illustrate this, below we include a portion of a letter that a friend of one author of this paper wrote to her husband when they were both in their 60s:

We often joke about our aging bodies—your baldness—the spider veins on my legs‐‐and the extra flesh we both carry around on our waistline. But over the years of our marriage I have come to see our bodies with new eyes. There is a beauty that comes from weaving two lives together and having a shared history. Our history together is literally written on our bodies. The lines on your face mark times of laughter, times of tears, and the depressions that we have walked through together. The scars on your knees remind me of your knee replacement surgery and our joy when you were able to once again enjoy hikes … My spider veins that occurred during pregnancy are vivid markers that God has blessed us with 4 amazing children. My old and worn hands speak of household projects: the brick patio, gardening, refinishing furniture, all efforts to create a home for you and the kids.^25^

Within our restructured banquet analogy, it could be more readily imagined why a person with a “normative” understanding of human nature might feel prepared for the banquet to come to an end; as the advantageous position of love is not without its mental, physical, and emotional costs.^26^ Given its benefits, it is reasonable to conclude that it is still a way of life worth adopting.^27^

Many of the costs associated with love are cumulative and not directly tied to the aging process–such as might be the case when caring for a loved one who is experiencing long‐term mental or physical ailments. But here we would also amend the analogy of a race. What we are generally proposing with a life oriented toward love is not a life of near‐constant exhaustion; but one where there is still room for rest and play. A better analogy than a race may then be a gentle, yet fairly persistent, walk up the gradual incline of a lovely mountain.

Additionally, in consideration of the meaning and significance found through an orientation of love, one could reasonably not want to forfeit or forgo that orientation for lengthy periods of time (presumably to rest and recover, both mentally and physically, before returning to service in the good of others for perhaps another 100 years). Here then we run into how to weigh goods against one another–especially as it relates to other goods one might forfeit by choosing to die at 100. The question of weighing goods leads us on to the question of what it means to live well, on which reasonable persons can reasonably disagree; and which cannot be reduced entirely to a normative understanding of human nature.

## 5 Avoiding Antagonism and Estrangement

Let us now take up one possible objection: Why not at least try life extension? You could always choose to die if it proved unsatisfactory. In response, we believe there are good reasons to dispute Linden's claim that widespread life extension will not engender overpopulation or other undesirable social outcomes, though we will not do so in detail here [24]. We will only say that one might reasonably conclude it is an act of necessary love to forgo the inhibition of aging so as to not risk the imposition of birth restrictions, or else transform society in other radical ways—especially without the full consent of any particular populace.^28^

Additionally, choosing contingent immortality, even on a trial basis, could subvert an orientation of love toward those closest to us. That is, a quest for mastery over our bodies might introduce an antagonism and encourage instrumentalization. Returning to the marriage example, consider the following kinds of conversation: “I am now 225. We have lived together for 190 of those years. It is not your fault, but I now feel that death is preferable to life with you. The good no longer outweighs the bad.” The same kind of bleak (and almost comical) conversation could be imagined between a parent and child, or even between dear friends.^29^ Consider also the tragedy of this kind of appeal: “Please don't choose to die! Am I not enough for you?”

It would also be mentally distressing and disorienting to watch potentially dozens of loved ones die (here we are assuming that not everyone will choose to live to the same age in Linden's imagined future, or even that everyone will opt for life extension). Would it not correspondingly become difficult to love, and so open your heart to another, after watching so many people die? Similarly, what would happen if one did definitively find life extension to be unpalatable? They, for example, might discover that the extended work of love (and even the securing of simple necessities, like working a job to make sure the bills are paid, etc.) felt more burden than blessing. In alignment with Lucretius’ banquet analogy, it would seem to us a very sad death to leave this life feeling disaffected and estranged from an orientation of love, or what one had previously experienced as a source of deep meaning and purpose.

### 5.1 Why 100?

We acknowledge that much of what we have argued in favor of a natural human lifespan could reasonably be applied to the proactive choice to limit one's number of years before the inhibition of aging. For example, one could choose a limit of 200 or 300 years. Such a number would still provide a person with a reference point for the “race” of love, could help prevent some potential antagonism and estrangement, and imbue acts of love with a particular and rich sense of meaning and purpose (“I am choosing to spend my 200 years on this earth loving in these particular ways”).

Nevertheless, a person could still make a reasonable choice in limiting themself to 100 years based on the above‐mentioned fears over the remaking of society. For example, job markets might become saturated–making it exceedingly difficult for younger people to find work that is profitable and meaningful to them. Additionally, one might sensibly conclude that 100 years is simply the number of years they believe they can reasonably consider love to be a benefit to them. After that, they might imagine again that it would feel more like a begrudging task than a glad benefit. For example, perhaps they plan a career entailing manual labor, and judgee 200 or 300 years of that labor, even when directed in part toward the love of others, to be more than they would care to undertake. As we mentioned, rigorous work, including of the emotional, physical, and mental kind, has a cumulative, holistic kind of toll on us as embodied beings that is not entirely equatable with the aging process.

Data cited by Linden also indicates that nearly 70% of Americans would want to die somewhere between the ages of 78 and 100 [3, p. 5]. Additionally, a naturally bounded lifespan is all we have collectively known as human beings. And at least some have found that boundedness compatible with a sense of having lived a meaningful and complete life. We know, then, such an experience is possible for ourselves. At least for now, we have no similar testimony from those who have lived well past a natural human lifespan. Returning to Sayers notion of experiential truth, it might then be argued that shared human experience points us toward embracing a natural lifespan of 100 years.

In summation, we believe there still are good reasons to choose a 100‐year life span, but, given the limited scope of our article, also concede that our narrower focus on love might reasonably allow for persons to proactively select for longer, definite lifespans.

## 6 Conclusion

Neither we nor Linden are advocating a permanent immortality. Linden himself doubts he will want to live forever [3, p. 20]. Given the choice between living to 100 years, and that of contingent immortality, it is a matter of when we will die, not if. What, then, is the best time to do so? Linden asks at the outset of his book: “Is it bad to die? Or is death after a long, full life a good, fitting end? Our answer to this question … reveals our attitudes about the purposes of life and our place in the cosmos” [3, p. 1]. We agree that the question does come down to conceptions of the purpose(s) of life. This article, we have focused on an experiential claim relating to self‐giving love: that is the experience of many people that living a life oriented toward love is a meaningful and purposeful endeavor. At the same time, a life of love is not without its costs. Drawing on that claim, it is our conclusion that death can in fact be a fitting end to a long, full life oriented toward self‐giving in service of the good of others.

## Data Availability

Data sharing is not applicable to this article as no data sets were generated or analyzed during the current study.

## Endnotes

1 Death would still be possible through murder, accidents, and so forth.

2 For further discussion on deprivationism, see Kagan [1]. For a response to Kagan (which focuses on the question of ending one's life, rather than explicitly life extension), see Kamm [2].

3 Given Linden's despisal of the aging process, we presume that the appeal of such a pill would be the inhibition, rather than reversal, of aging. Meaning one would want to inhibit aging around their physical peak, rather than living in accordance with the natural aging process to 50, for example, and then reverting back to a biological age of 25.

4 Here our claim is not normative, in the sense that one would be morally obligated to limit their life as such. Instead, we set out to demonstrate that a person would not be unreasonable or foolish for doing so.

5 In line with the thinking of Linden, we do not consider the idea of transferring one's mind to a computer, as we deem it a metaphysical impossibility.

6 For discussion, see Gavrilova and Gavrilov [5].

7 For example, see Lu et al. [6].

8 For a short discussion of several proposals, see Linden [3, pp. 3–4].

9 For a related discussion, see Abad et al. [7].

10 Linden [3, p. 20]. It is not clear to us how inhibiting aging would remove the threat of death from all diseases, notably those of infectious origin.

11 We use the term “live well” to refer to what a person might deem a superior way of being—or a way of experiencing, relating to, and interacting with oneself, other persons, the natural world, and self‐recognized supernatural realities. Questions pertaining to living well need to be answered partly in the light of conceptions of the shared nature of human beings, but also our purpose or purposes as creatures, our place in the natural order, and attending conceptions of the highest forms of flourishing.

12 For a discussion by Linden on these matters, see Linden [3, Ch. 5].

13 For an overview of relevant literature, see Glass et al. [15]. Here we would distinguish the happiness often identified in such surveys from how St. Augustine spoke of it. Namely, as joy grounded in truth (*Confessions*, Book X).

14 For an overview of relevant literature, see Gold et al. [16].

15 We also believe that even if no sense of meaning or purpose were derived from self‐giving in the service to others, it would still constitute a worthwhile undertaking because of the inherent value of other persons.

16 The latter quotation was originally found in Snead [9, p. 88].

17 Such limitations are implied by the reference to “self‐giving” in our definition of love.

18 In contrast, it could be imagined that the bishop's sister was not aware of any particular, natural limitations as she went about loving the bishop—yet still found meaning and purpose through that orientation of love. A young child also provides an example of a person who could find meaning through an act of love, yet not necessarily be aware of relevant limitations.

19 The professor might say, for example, that one purpose of my time‐bound life is to serve the good of my students, and similarly one purpose of any particular day is to do the same.

20 For a related discussion on love as a kind of ongoing project that unfolds over time and works toward specific goals, see Nussbaum [18]. Nussbaum also suggests that awareness of the bounded nature of the human lifespan can imbue our decisions in life with a special sense of meaning and purpose (a position she later amended).

21 Here we do not consider the possibility of planning to intentionally shorten one's natural lifespan, such as committing suicide at age 40, so as to an attain a special sense of meaning or significance. We would reject such a practice on the grounds of the inherent value of the individual, embodied human person. In contrast, we hold the human lifecycle to be a constitutive part of shared and embodied human existence, and so it does not contravene that inherent value.

22 We would question some aspects of this claim of contingency—and specifically suggest that the underlying “genetic programs” that help guide the development and design of our bodies might inhibit a longer and natural average human lifespan. We would then add that the natural, average human lifespan, given our present human form, may already be, or nearly be, “maxed out” in some developed nations. For further discussion, see Olshansky [19].

23 Nussbaum [18, p. 203]. Reversing a former position, and in agreement with John Martin Fischer, Nussbaum assesses elsewhere that an eternal kind of banquet is not necessarily undesirable. Drawing upon the banquet analogy, a person could take a break from eating and revelry and pursue other enjoyable activities, after which banquet matters might resume. See Nussbaum [21].

24 For critique of the notion that “we need to believe our days are numbered to live well,” and other ways mortality is said to imbue our actions with a sense of meaning and significance, see Linden [3, Chs. 6–7].

25 Private correspondence, shared with A. Moeller.

26 Our argument draws upon, but differs, from that of Bernard Williams on boredom and immortality. We are not asserting that an orientation of love, if it were to extend well past 100 years, would necessarily lead to boredom. Instead, we hold that our bounded or limited natures as embodied beings could result in the experience of disaffectedness toward an orientation of love, given the associated costs. See Williams [23].

27 We acknowledge little has been said about the difficulties associated with the aging process. We are not able to anticipate all of the ailments an aging person might face in the year 2300, no their severity. Still, we believe that chronic pain, poor eyesight, etc., can be fully acknowledged as difficult, and reasonably be thought of as a constitutive part of leading a meaningful and purposeful life of love. At the same time, we think it is generally a very good thing to help alleviate the pain, suffering, and discomfort associated with aging.

28 For example, even if one inhibits aging at 25, but still chooses to die at 100 upon being dissatisfied with that inhibition, it would seem likely that women would give birth to more children—given the avoidance of menopause.

29 Of course, a person might also say: “I need an extremely long break from you, so that at a later point I can come back to love you. It is simply too much to continue for so long.” But we would still hold this to be a strange affront to an orientation of love.
